# Supplementary material for: Renin-angiotensin system inhibitors and susceptibility to COVID-19 in patients with hypertension: a propensity score-matched cohort study in primary care
Source: BMC Infect Dis. 2021 Mar 15;21:262. doi: 10.1186/s12879-021-05951-w (PMC7957446; doi:10.1186/s12879-021-05951-w)
Supplement: Supplementary file 1 — Additional file 1: Supplementary Table 1. Read codes used for the ascertainment of the primary outcome (confirmed/suspected COVID-19) [file 12879_2021_5951_MOESM1_ESM.docx]

**Supplementary Table 1: Read codes used for the ascertainment of the primary outcome (confirmed/suspected COVID-19)**

| **Code** | **Description** |
| --- | --- |
| G558500 | Cardiomyopathy due SARS-CoV-2 |
| A795200 | COVID-19 confirmed by laboratory test |
| G520800 | Myocarditis due to SARS-CoV-2 |
| H051100 | URTI due to SARS-CoV-2 |
| A076400 | Gastroenteritis due to SARS-CoV-2 |
| F529.00 | Otitis media due to SARS-CoV-2 |
| F289.00 | Encephalopathy due to SARS-CoV-2 |
| A795300 | COVID-19 confirmed using clinical diagnostic criteria |
| H204.00 | Pneumonia due to SARS-CoV-2 |
| 43hF.00 | Detection of SARS-CoV-2 by PCR |
| 4J3R100 | 2019-nCoV (novel coronavirus) detected |
| 9N31200 | Telephone consultation for suspected 2019-nCoV (novel coronavirus) |
| A795100 | Disease caused by 2019-nCoV (novel coronavirus) |
| 1JX1.00 | Suspected disease caused by 2019-nCoV (novel coronavirus) |
| 1JX..00 | Suspected coronavirus infection |
| 43dt400 | Has immunity to SARS-CoV-2 |
| 43dtA00 | SARS-CoV-2 IgG detected |
| 43dtG00 | SARS-CoV-2 IgM detected |
